# Cost-effectiveness of Universal and Targeted Hepatitis C Virus Screening in the United States

**DOI:** 10.1001/jamanetworkopen.2020.15756

**Published:** 2020-09-03

**Authors:** Moosa Tatar, Susana W. Keeshin, Mark Mailliard, Fernando A. Wilson

**Affiliations:** 1 Matheson Center for Health Care Studies, the University of Utah, Salt Lake City; 2 Division of Infectious Disease, the University of Utah School of Medicine, Salt Lake City; 3 University of Nebraska Medical Center College of Medicine, Omaha

## Abstract

**Importance:**

Between 2 and 3.5 million people live with chronic hepatitis C virus (HCV) infection in the US, most of whom (approximately 75%) are not aware of their disease. Despite the availability of effective HCV treatment in the early stages of infection, HCV will result in thousands of deaths in the next decade in the US.

**Objective:**

To investigate the cost-effectiveness of universal screening for all US adults aged 18 years or older for HCV in the US and of targeted screening of people who inject drugs.

**Design, Setting, and Participants:**

This simulated economic evaluation used cohort analyses in a Markov model to perform a 10 000-participant Monte Carlo microsimulation trail to evaluate the cost-effectiveness of HCV screening programs, and compared screening programs targeting people who inject drugs with universal screening of US adults age 18 years or older. Data were analyzed in December 2019.

**Exposures:**

Cost per quality-adjusted life-year (QALY).

**Main Outcomes and Measures:**

Cost per QALY gained.

**Results:**

In a 10 000 Monte Carlo microsimulation trail that compared a baseline of individuals aged 40 years (men and women) and people who inject drugs in the US, screening and treatment for HCV were estimated to increase total costs by $10 457 per person and increase QALYs by 0.23 (approximately 3 months), providing an incremental cost-effectiveness ratio of $45 465 per QALY. Also, universal screening and treatment for HCV are estimated to increase total costs by $2845 per person and increase QALYs by 0.01, providing an incremental cost-effectiveness ratio of $291 277 per QALY.

**Conclusions and Relevance:**

The findings of this study suggest that HCV screening for people who inject drugs may be a cost-effective intervention to combat HCV infection in the US, which could potentially decrease the risk of untreated HCV infection and liver-related mortality.

## Key Points

**Question:**

Are universal hepatitis C virus (HCV) screening for all US adults aged 18 years or older and targeted HCV screening among people who inject drugs cost-effective for limiting HCV infection?

**Findings:**

In this economic evaluation study, HCV screening for people who inject drugs in the US increased quality-adjusted life-years (QALYs) by 0.23 (ie, approximately 3 months), with an incremental cost-effectiveness ratio of $45 465 per QALY. Universal HCV screening increased QALY overall by 0.01, with an incremental cost-effectiveness ratio of $291 277 per QALY.

**Meaning:**

The findings of this study suggest that HCV screening for people who inject drugs may be a cost-effective intervention to combat HCV infection, which would decrease the risk of untreated HCV infection and liver-related mortality.

## Teaser

This economic evaluation analyzes the cost-effectiveness of 2 hepatitis C screening programs, a program targeting people who inject drugs and a universal program for US adults, and compares outcomes with the status quo in a Monte Carlo microsimulation.

## Introduction

The exact number of individuals in the US who are currently infected with hepatitis C virus (HCV) (presence of HCV RNA) is unknown, but it is estimated to be more than 2 million people and as many as 3.5 million people.^1,2^ Most individuals with HCV infection (approximately 75%) are not aware of their infection because few symptoms are evident in the early stages of the disease.^3,4^ However, 70% to 85% of acute HCV infections become a chronic disease.^5^ Chronic HCV infection is the primary reason for liver cirrhosis and hepatocellular carcinoma and the leading cause of liver transplantation.^6^ HCV causes nearly 40% of all chronic liver disease and is among the most common indications for liver transplantation in the US.^7,8^ From 2003 to 2014, 20 782 adults with chronic HCV underwent liver transplantation.^8^ HCV infection accounted for approximately 18 000 deaths in the US in 2016,^9,10^ and HCV infection–related mortality exceeds all other deaths from infectious diseases combined.^11^

Most people with HCV in the United States are individuals born between 1946 and 1964 (ie, the Baby Boomers).^12^ However, due to the slow progression of hepatitis C disease, the disease may remain undiagnosed for decades.^12^ Infection rates among persons who inject drugs (PWID) range from 30% to 90%, depending on frequency and duration of use, and account for approximately 60% of all HCV cases in the US.^13,14,15,16,17^ In 2011, the number of adults and adolescents (US population aged 13 years or older) who had injected drugs in their lifetime was reported to be approximately 6.6 million people.^18^ Therefore, while rates of HCV are higher among an older population, the US opioid epidemic has led to an evolving epidemiology of HCV.^4^ Sharing needles among PWID is a key risk factor for HCV transmission in US prisons and jails.^19^ Inmates in correctional institutions account for up to one-third of all US hepatitis C cases.^20,21^ Compared with a 1% infection rate for the general US population, HCV infection rates are particularly high in correctional institutions and range from 17.4% to 23.1%.^20,21^ One in 10 million people who pass through correctional settings each year have an undiagnosed HCV infection; more than 90% of these individuals are released to the general population.^22,23,24^ Throughout, these individuals may have little contact with the health care system, and as a result play a prominent role in the spread of HCV in US communities.^23,24,25,26^

HCV treatment can be very effective, especially if HCV is diagnosed in the early stages of the infection.^27^ Existing research shows that universal HCV screening in developed countries is effective.^28,29,30^ Prior studies on the cost-effectiveness of HCV screening in the US examined voluntary screening, specific population groups (eg, individuals born in the Baby Boomer generation, women experiencing pregnancy, volunteers for blood donations, and screening performed in US primary care settings), or older forms of treatment.^31,32,33,34,35^ However, few studies that have assessed the cost-effectiveness of HCV screening in the US account for recent and highly effective treatment regimens for HCV. Furthermore, the cost of new drugs is relatively lower than older drugs; prices for HCV treatments have decreased by approximately half. In the absence of such knowledge, the cost-effectiveness of universal and targeted screening for HCV remains uncertain.

The primary aim of this study was to assess and compare the cost-effectiveness of targeted screening for people who inject drugs with a universal HCV screening program for US adults aged 18 years or older, considering the most effective and recent medical treatments for HCV.

## Methods

This study was conducted based on structured reporting of economic evaluations of health interventions according to Consolidated Health Economic Evaluation Reporting Standards (CHEERS) guideline.^36^ CHEERS was followed in study assumptions and for reporting the cost-effectiveness analysis of universal HCV screening and targeted screening for PWID. The Common Rule exempts this study from institutional board review because no human participants were involved.

### Natural History of HCV

**Figure 1.**
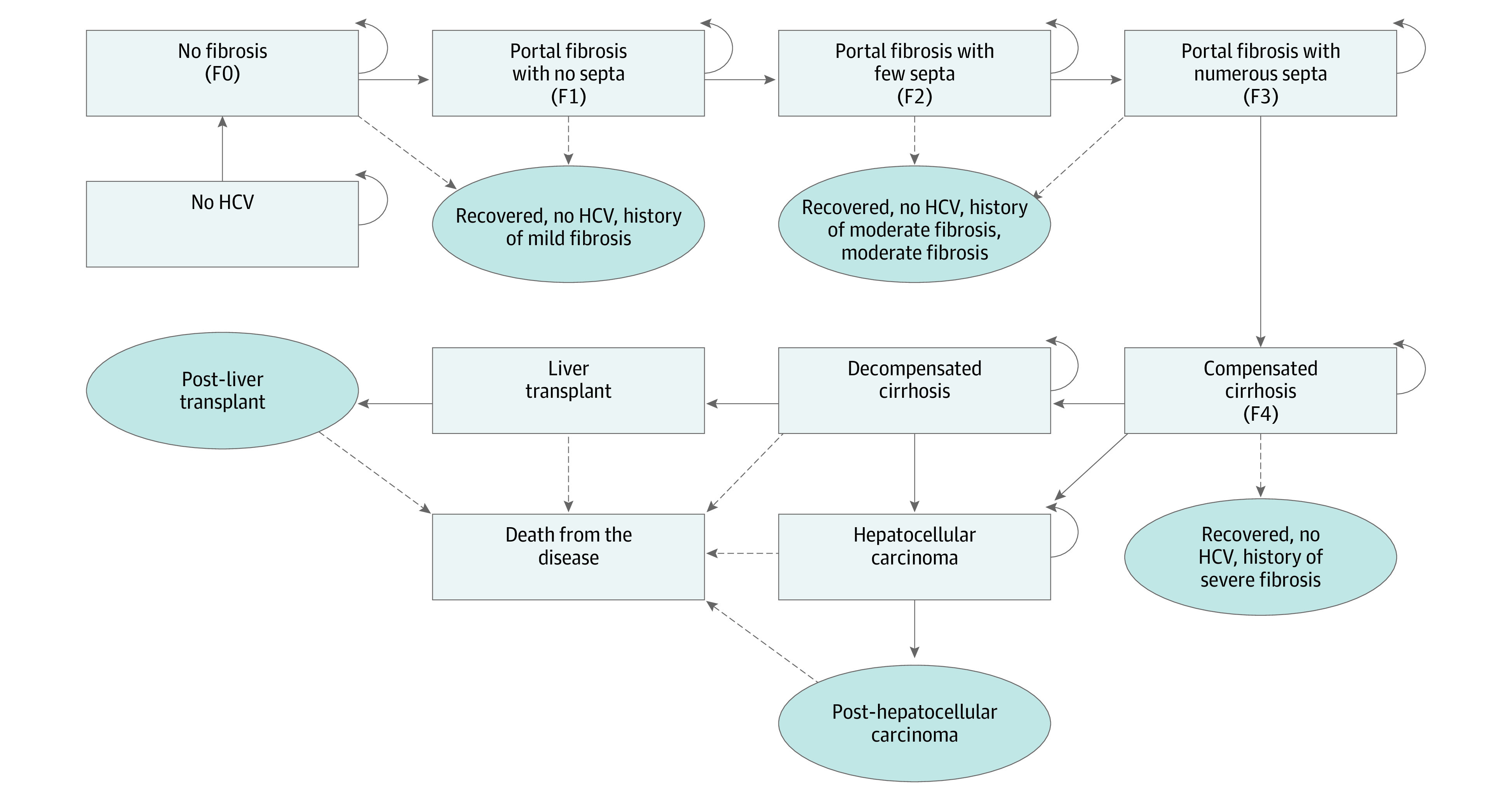
Markov Model of the Health State Transitions of HCV Health states are shown by rectangles, and have been determined according to the METAVIR liver biopsy staging system (F0-F4). Arrows show the transition between health states. Dotted arrows depict potential transitions between health states after treatment. Natural death also can happen in all states. HCV indicates hepatitis C virus.

This study was conducted using a decision-analytic Markov model of the natural history and progression of HCV to evaluate the cost-effectiveness of screening for HCV in the US population. The Markov model used in this study, based on the natural history of HCV according to empirically calibrated models, clinical characteristics, and published literature,^37,38,39^ shows disease progression for a person with HCV infection using a 10-year horizon (Figure 1). The base case at the time of screening and diagnosis was considered 40 years, which is approximately the median age in the US.

Disease progression was described in terms of METAVIR score, a liver biopsy staging system that assesses the severity of liver fibrosis.^40^ METAVIR scores assess the degree of scarring or fibrosis of the liver, ranging from F0 (no fibrosis) to F4 (cirrhosis). Health states were categorized in 2 main phases of disease progression: fibrosis progression stages F0 to F4, and nonfibrosis progression after stage F4. Health states included healthy (no HCV), no fibrosis (F0), portal fibrosis with no septa (F1), portal fibrosis with few septa (F2), numerous septa without cirrhosis (F3), compensated cirrhosis (F4), decompensated cirrhosis, hepatocellular carcinoma, post–hepatocellular carcinoma, liver transplantation, and post–liver transplantation.^40,41,42^ Without treatment, patients in the F0 state might experience spontaneous clearance of the HCV and return to a no HCV state in state F0. Otherwise, patients may progress to the next severity state of the disease (Figure 1). Patients who receive treatment and achieve sustained virologic response will transition to recovered states based on fibrosis severity. Patients with compensated cirrhosis who do not receive treatment will transition to hepatocellular carcinoma or decompensated cirrhosis and may receive liver transplantations. Because natural death could occur while a patient is in any state, likelihood of natural death in the Markov model is based on US life tables.^43^ Death from the disease can occur in the final nonfibrosis progression states. People may be reinfected at the same rate (1%) as the general population during and after successful treatment and, if reinfected,^44^ will begin fibrosis progression from the F0 state. The initial fibrosis state distribution was based on existing literature.^45^ The fibrosis transition rates (Table 1) vary with age and were based on published literature for HCV in the US, with possible transitions occurring every 12 months.^37,39,44^

**Table 1.** Transition Probabilities, Mortality Rates, and Costs

Variable	Probability	Source
**Transition state annual probabilities**		
Fibrosis progression, y		
<40	0.04	Saab et al,^44^ 2016
40-49	0.04	Liu et al,^39^ 2012
50-59	0.09	Liu et al,^39^ 2012
60-69	0.155	Liu et al,^39^ 2012
70-79	0.2	Liu et al,^39^ 2012
≥80	0.285	Saab et al,^44^ 2016
Nonfibrosis progression		
Recovered (SVR), no HCV, history of mild or severe fibrosis (compensated cirrhosis) to hepatocellular carcinoma	0.012	Saab et al,^44^ 2016
Compensated cirrhosis to decompensated cirrhosis	0.04	Liu et al,^39^ 2012
Compensated cirrhosis to hepatocellular carcinoma (first year)	0.02	Liu et al,^39^ 2012
Decompensated cirrhosis to hepatocellular carcinoma (first year)	0.02	Liu et al,^39^ 2012
Liver transplantation		
Decompensated cirrhosis to transplantation (first year)	0.05	Liu et al,^39^ 2012
Hepatocellular carcinoma to transplantation (first year)	0.15	Liu et al,^39^ 2012
Liver-related mortality		
Decompensated cirrhosis to liver death	0.26	Liu et al,^39^ 2012
Hepatocellular carcinoma (first year) to liver death	0.72	Liu et al,^39^ 2012
Hepatocellular carcinoma (subsequent year) to liver death	0.25	Liu et al,^39^ 2012
Transplantation to liver death	0.14	Liu et al,^39^ 2012
After transplantation to liver death	0.05	Liu et al,^39^ 2012
Viral reinfection	0.01	Saab et al,^44^ 2016
Spontaneous remission from no fibrosis	0.012	Liu et al,^39^ 2012
Mortality rate total population, y		
<40	0.001	Arias and Xu,^43^ 2019
40-49	0.002	Arias and Xu,^43^ 2019
50-59	0.006	Arias and Xu,^43^ 2019
60-69	0.012	Arias and Xu,^43^ 2019
70-79	0.026	Arias and Xu,^43^ 2019
≥80	0.186	Arias and Xu,^43^ 2019
**Costs, $**		
Direct medical annual costs by health state		
No HCV	0	Assumed
Screening	140	Carlson,^50^ 2005
Glecaprevir and pibrentasvir regimen (8 wk)	26 400	Wholesale acquisition costs,^47^ 2017
Decompensated cirrhosis treatment	33 314	McAdam-Marx et al,^48^ 2011
Hepatocellular carcinoma treatment		
First year	52 248	McAdam-Marx et al,^48^ 2011
Subsequent years	52 248	McAdam-Marx et al,^48^ 2011
Liver transplantation		
First year	812 000	Bentley et al,^37^ 2003
Subsequent years	45 481	McAdam-Marx et al,^48^ 2011
Variable, value (range)		
Reinfection rate	0.01 (0.01-0.10)	NA
Infection rate for general population	0.01 (0.01-0.10)	NA
Infection rate for people who inject drugs	0.60 (0.30-0.90)	NA
Cost of HCV treatment (drug), $	26 400 (2640-52 800)	NA

Abbreviations: HCV, hepatitis C virus; NA, not applicable; SVR, sustained virologic response.

### Treatment

The treatment regimen included in this analysis was a combination of glecaprevir and pibrentasvir, used to treat chronic HCV genotypes 1, 2, 3, 4, 5, or 6 without cirrhosis or with compensated cirrhosis. The treatment protocol is 3 tablets (100 mg glecaprevir and 40 mg pibrentasvir) taken daily for 8 weeks. The treatment is highly effective, with a success rate of 98%.^46^ The regimen is not effective for people with advanced cirrhosis (ie, decompensated cirrhosis).

### Cost

Direct medical costs related to HCV included in the study are listed in Table 1. Drug costs in this study were based on wholesale acquisition cost, which is an estimate of the manufacturer’s list price and does not include discounts or rebates, and covered the duration of the treatment.^47^ Health state medical costs, which are the cost of treatment based on the severity and progress of the disease, were obtained from the published literature on an annual timeframe.^37,48,50^ Costs considered included visits (inpatient and outpatient), diagnostic and laboratory testing, physician services, emergency department place of service, and pharmacy.^44,48^ The total cost for liver transplantation was obtained from US organ and tissue transplantation cost estimates provided by a prior study.^49^ The cost of an HCV screening test was assumed to be $140 based on the existing literature.^50^ Finally, the cost of no HCV was assumed to be $0. Costs were inflation-adjusted to 2019 US dollars using the US Consumer Price Index, when necessary.^51^

### Statistical Analysis

The primary outcome of this study was quality-adjusted life-years (QALYs) gained, and incremental cost-effectiveness ratios (ICERs) were calculated. Costs were discounted by 3% annually based on recommendations from the US Panel on Cost-effectiveness in Health and Medicine.^52,53^ Life tables from the US Centers for Disease Control and Prevention (CDC) were used for natural mortality rates for the US population.^43^ This study was conducted in 2019, and data were analyzed in December 2019. The baseline of analysis was a 40-year old individual in the United States. Cost-effectiveness analysis was conducted using a societal perspective to assess and compare the status quo with screening for HCV under 2 scenarios: annual screening of the total population (universal screening) and targeted screening of PWID. ICERs were calculated by dividing the difference in expected costs of each scenario by the difference in their effectiveness (ie, QALY). The targeted screening scenario is based on a probability of 60% HCV infection in the PWID population vs a probability of 1% under the universal screening scenario.^54^ HCV infection rates for PWID depend on frequency and duration of use and range from 30% to 90%.^13,14,15,16,17^

To determine the expected rates of liver-related mortality, HCV infection, and liver transplantation in the 2 screening scenarios, a 10 000 trial Monte Carlo microsimulation was undertaken. A Monte Carlo microsimulation performs repeated random sampling to get results in a process that cannot easily be estimated and performed. The Monte Carlo microsimulation used in this study estimated the effects of screening if the HCV screening program were scaled to 10 000 people. The microsimulation calculated the results 10 000 times, each time using a different set of random values from the model probability functions to calculate overall totals of liver-related mortality, HCV infection, and liver transplantation for a sample of 10 000 people in a 10-year horizon.

Sensitivity analyses were conducted to measure and evaluate the uncertainty derived from the model assumption. Sensitivity analyses recalculated outcomes under alternative assumptions and used a range of probabilities and values instead of a specific probability and value to determine the association of a variable (ie, reinfection rate, infection rate for both the general population and PWID, cost of HCV treatment) with the outcomes. The TreeAge Pro Healthcare 2019 (TreeAge Software Inc) statistical package was used for all analyses.

## Results

Table 2 presents ICERs of costs to QALYs gained for PWID-targeted screening and universal screening compared with the status quo determined by Markov modeling. The PWID screening scenario is based on a probability of 60% HCV infection of this population. In Table 2, the cost per QALY of PWID screening is $2311.50 vs $915.70 for the status quo. Relative to the status quo, screening and treatment of the PWID population is estimated to increase total costs by $10 457 per person on average for a 10 000 Monte Carlo microsimulation trail, resulting in an increase in QALYs of 0.23 (approximately 3 months) during their expected lifetime. The ICER in this scenario is estimated to be $44 815 per QALY gained, which is smaller than $50 000 per QALY value.^55^ This means that this scenario is cost-effective.

**Table 2.** Incremental Cost-effectiveness Ratios of Costs to QALYs Gained for PWID and Universal Screening vs Status Quo Using Markov Modeling

Strategy	Cost, $	Incremental cost, $	Effectiveness, LY	Incremental		Cost-effectiveness,^a^ $/LY
				Effectiveness, LY	Cost/effectiveness, $	
No HCV screening (status quo) vs HCV screening for PWID						
Status quo	6507	NA	7.11	NA	NA	915.7
PWID screening	16 963	10 457	7.34	0.23	44 815	2311.5
No HCV screening (status quo) vs HCV screening for all US adults ages ≥18 y						
Status quo	108	NA	7.51	NA	NA	14.4
Universal screening	2953	2845	7.52	0.01	29 1277	392.7

Abbreviations: HCV, hepatitis C virus; QALY, quality-adjusted life-year; NA, not applicable; PWID, persons who inject drugs.

^a^ Incremental cost-effectiveness is the difference in cost divided by difference in effectiveness between PWID screening and status quo.

Table 2 also presents the results of universal screening of all US adults in which 1% of the population have HCV infection.^54^ The cost per QALY of universal screening is $392.70 vs $14.40 for the status quo. Relative to the status quo, HCV infection for the total US population is estimated to increase total costs by $2845 and shows a very small increase in QALYs of 0.01. The ICER for universal screening is estimated to be $291 277 per QALY gained, which is greater than $50 000 per QALY value. This means this scenario is not cost-effective.

Results from a 10 000 trial Monte Carlo microsimulation analysis showed that PWID screening vs status quo is estimated to reduce liver-related mortality by 88 deaths and new infections by 8754. In addition, the model estimated that the number of liver transplantations decrease by 18 during a 10-year horizon. Results from a 10 000 trial Monte Carlo microsimulation analysis showed that universal screening vs the status quo is estimated to reduce liver-related mortality by 1 death and new infections by 3053 during a 10-year horizon.

**Figure 2.**
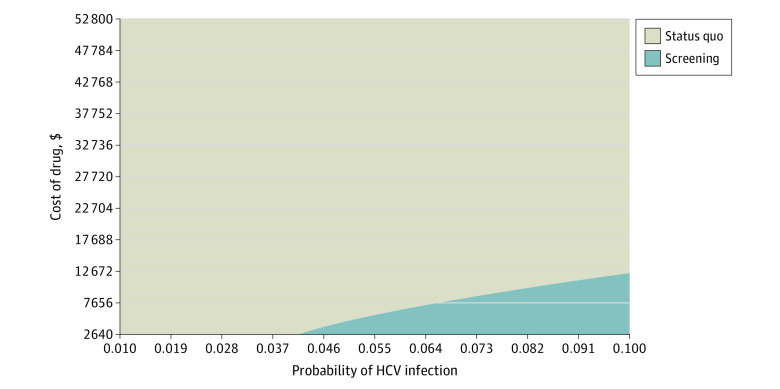
Sensitivity Analysis of the Probability of HCV and Cost of the HCV Drug Treatment for Universal Screening HCV indicates hepatitis C virus.

Results from the 2-way sensitivity analysis for PWID showed that, based on the assumption that 60% of PWID have HCV infection and the reinfection rate is 1%, the cost of the HCV drug treatment could increase from $26 400 to $29 054 per PWID and ICER would still be less than $50 000 per QALY, meaning HCV screening would still be cost-effective (Figure 2). Additionally, HCV screening for PWID is estimated to be cost-effective over a broad range of HCV infection rates. Given the HCV drug treatment cost of $26 400 per patient, the HCV infection rate could decrease to 40% and ICER would still be less than $50 000 per QALY; HCV screening would still be cost-effective. Moreover, based on the assumption that 60% of the PWID have HCV infection and that HCV drug treatment costs $26 400 per patient, the reinfection rate could increase to 3.5% and the ICER would still be less than $50 000 per QALY and HCV screening would still be cost-effective.

Universal HCV screening could be cost-effective across a range of HCV infection rates and costs of HCV drug treatment. For example, if the cost of the HCV drug treatment decreased from $26 400 to $13 200 per patient and the infection rate increased to 10%, the ICER would be less than $50 000 per QALY and HCV screening would still be cost-effective. Also, if the infection rate increased to 4% and the cost of the HCV drug treatment decreased to $2640 per patient, the ICER would be less than $50 000 per QALY and HCV screening would still be cost-effective.

**Figure 3.**
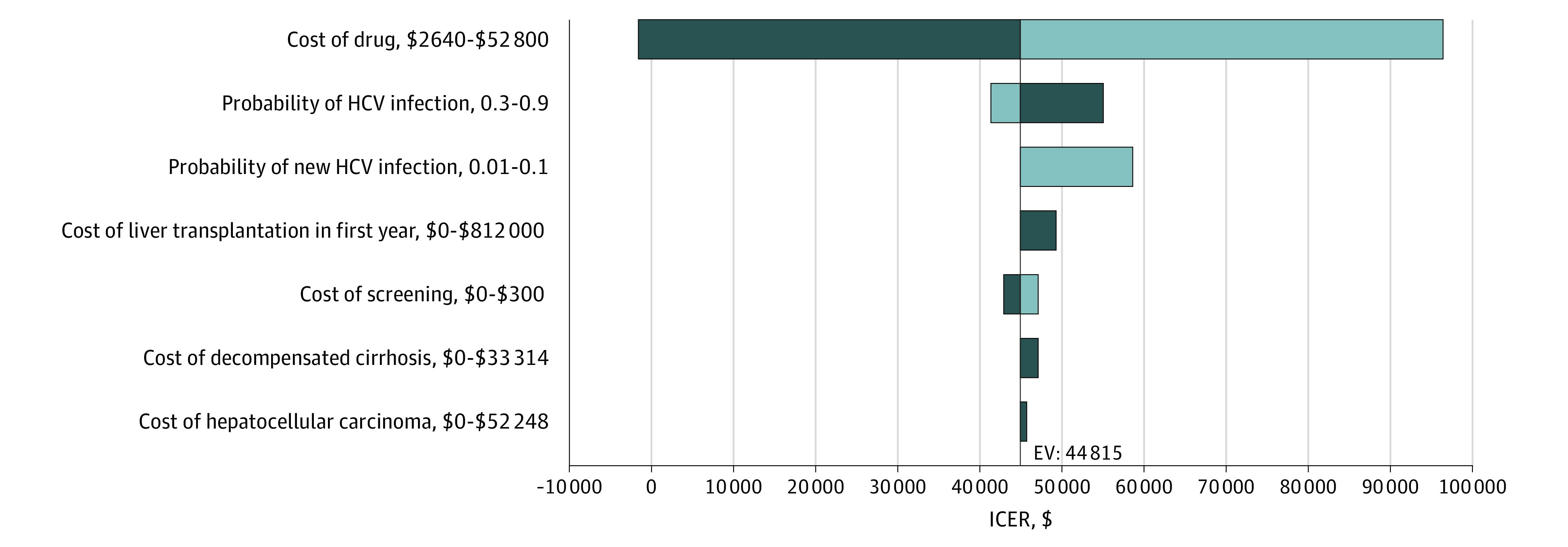
Associations of Variation in HCV Infection, Cost of Drug Treatment, Probability of New HCV Infection, and Medical Treatment Cost by Stage of Disease The dark blue portion of each bar represents the low range of the parameter listed on the y-axis, and the light blue portion of the bar represents the high range of the parameter. When dark blue is on the left and light blue on the right, the ICER increases as the parameter value increases; when light blue is on the left of the baseline, ICER decreases as parameter value increases. EV indicates expected value; HCV, hepatitis C virus; ICER, incremental cost-effectiveness ratio.

Figure 3 depicts the associations of independent variations in the probability of HCV infection, the cost of the HCV drug treatment, the probability of a new HCV infection, and the cost of medical treatment by stage of disease. The result of the tornado diagram indicates that the cost of the HCV drug treatment and the probability of HCV infection are the most influential parameters in the model.

## Discussion

This study examined the cost-effectiveness of universal HCV screening and targeted screening of PWID adult populations for the US using Monte Carlo microsimulation analysis. This analysis used recent data on effectiveness and costs of the pangenotypic regimen of glecaprevir and pibrentasvir as treatment for chronic HCV, which costs approximately half the price of older treatments. Our results showed that HCV screening for PWID is cost-effective with an ICER of $44 815 per QALY. Furthermore, Monte Carlo microsimulation results for a PWID population showed that screening and treatment were associated with prevention of at least 88 HCV-related deaths and reduction in new infections by 8754 cases relative to the status quo. The proposed strategy was associated with avoidance of HCV-related deaths and new infections by 99% and 83%, respectively. In this scenario, screening PWID for HCV was associated in a reduction of the number of liver transplantations by 18. However, universal screening for the total adult US population was not cost-effective in our analysis.

Our study findings for PWID-targeted HCV screening were consistent with results of screening of high-risk populations reported in other countries, including the Netherlands, Canada, Japan, and the United Kingdom.^28,29,30,56^ Previous studies of HCV screening in the US are mixed. HCV screening is reported to be cost-effective when the prevalence of HCV is high in the total population or in primary care settings.^31,32^ Some studies did not support widespread screening for HCV for average-risk adults.^34^ In contrast, other studies suggested that broad screening for the general population can be cost-effective.^57,58^ For example, Eckman et al^58^ reported that 1-time universal screening of adults was cost-effective in increasing quality-adjusted QALYs. However, the treatment cost for the advanced stages of the disease was considerably lower, and the drug regimen examined was different and substantially lower in cost than that of the glecaprevir and pibrentasvir regimen examined in our study. A 2018 study^59^ from the US Preventive Services Task Force (USPSTF) recommended screening for HCV infection in adults aged 18 to 79 years.^13^ However, the USPTF recommendation is based on the effectiveness of screening on improving health outcomes and not on a cost-effectiveness analysis.

Most prior studies that analyzed the cost-effectiveness of HCV screening used data on specific populations or older pharmaceutical treatment protocols than what are currently available.^31,32,33,34,35^ Few studies have assessed the cost-effectiveness of HCV screening in the US considering recently introduced effective treatments of HCV.

In our study, a combination of glecaprevir and pibrentasvir was examined as the drug treatment of early stage HCV. It is a highly effective regimen that is low cost relative to older drug protocols, costing $26 400 for an 8-week treatment period. However, despite the lower cost, screening of all US adults was not found to be cost-effective in our analysis, resulting in an ICER of nearly $300 000 per QALY.

Our study also analyzed the cost-effectiveness of targeted HCV screening for the PWID population. The prevalence of HCV infection among PWID is very high, having been estimated to be between 30% to 90%.^13,14,15,16,17^ Addressing HCV infection among PWID by diagnosing and treating infection in its early stages can prevent many HCV-related complications and deaths in the US and substantially decrease health care expenditures. Our study did not examine how PWID screening may be best accomplished. For example, routine screening of incarcerated populations may be effective.^22^ However, ineffective or costly screening programs of PWID will limit the potential cost-effectiveness that we report in our analysis.

### Limitations

Our study has limitations. In the Markov model, potential HCV reinfection was assumed to be 1%, which is the probability of infection for the general population. However, HCV reinfection rates are higher among PWID (11%).^60^ Furthermore, our model included only direct medical costs. Indirect costs related to HCV treatment may be significant, such as patient mental health status and caregiver costs. We used wholesale acquisition costs, which are frequently used in economic analysis because they are reasonably transparent and consistent.^61^ Direct cost estimates were obtained from published sources and may not fully reflect the actual costs of treatment for a particular region or medical system within the US. Finally, our modeling assumes individuals complete the 8-week drug treatment regimen if diagnosed. To the extent that individuals fail to complete the regimen, our analysis may overstate the cost-effectiveness of screening.

## Conclusions

Drug injection is a key risk behavior for HCV infection, resulting in an infection rate among PWID of 30% to 90%. If untreated, HCV infection may progress to liver cirrhosis and, ultimately, death. New drug protocols have been developed to successfully treat HCV, and this study examines the cost-effectiveness of targeted screening of PWID and universal screening for the US adult population. Results from our analysis, using an ICER of $50 000 per QALY as a cutoff, suggest that HCV screening that is tailored to PWID is cost-effective in averting premature deaths and liver transplantations associated with HCV disease progression.
